# An Interesting Case of Prostate Cancer Presenting With Colonic Metastasis

**DOI:** 10.7759/cureus.34602

**Published:** 2023-02-03

**Authors:** Shawn Keating, Ayesha Imtiaz, Kenneth Nahum, Ankita Prasad, Pramil Cheriyath

**Affiliations:** 1 Internal Medicine, Hackensack Meridian Health, Ocean Medical Center, Brick, USA; 2 Oncology, Hackensack University Medical Center, Brick, USA; 3 Internal Medicine, Ocean University Medical Center, Brick, USA

**Keywords:** colonic metastasis, prostate cancer, prostate carcinoma, liver metastasis, prostate-specific antigen, psa, rectal metastasis, adenocarcinoma, metastatic prostate carcinoma

## Abstract

Prostate cancer is common cancer that grows slowly and tends to metastasize to bones, lungs, and the liver. Most malignancies have established patterns in presentation, localization, and organs where they metastasize. We are presenting a case of a 60-year-old man who presented with abdominal pain and, on further investigation, was found to have polyps in the colon, a flat rectal mass with eccentric thickening of the rectum, a moderately enlarged prostate, and multiple liver masses suggestive of metastasis. It was initially thought to be colorectal cancer with metastasis but was eventually diagnosed as a stage IV prostate adenocarcinoma with metastases to the liver and rectum. It is very unusual for prostate cancer to present with distal metastasis to the liver and rectum, as in this case.

## Introduction

Prostate cancer is one of the most common malignancies affecting men in the United States. An estimated 11% of Americans will have the disease in their lifetime [1], with the incidence generally rising with age. The known major risk factors are age, ethnicity, obesity, and family history. Most of the time, prostate cancer is adenocarcinoma, which starts in the organ's glands. It is usually slow growing, with a typical five-year survival rate of 98% [1]. However, metastatic prostate carcinoma has a poor prognosis with a five-year survival rate of 30.5% [1]. It usually metastasizes to the axial skeleton, followed by the lungs and liver.

## Case presentation

Our patient is a 60-year-old male who presented with abdominal pain for two weeks. The pain was described as a dull ache in the epigastric and right upper quadrant areas, accompanied by nausea and vomiting. It was not worsened by food or activity, and he received some antacids and pantoprazole, but they did not help his pain. He did not have a change in bowel or bladder habits, and there was no history of blood in vomitus or stool. He was not taking painkillers and had not had a similar episode in the past. There were no other significant past medical or surgical histories. He had not seen the doctor in years and had no screening tests. He had a family history of lung cancer in both parents and was a non-smoker, drinking two to four beers per month. He worked as a mechanic for ambulances.

At presentation, he was conscious and alert with stable vitals, a temperature of 98.7° Fahrenheit, blood pressure of 136/74 mm Hg, pulse rate of 75 beats per minute, a respiration rate of 17 breaths per minute, and oxygen saturation of 99% on room air. Physical examination at the presentation showed no abnormalities, and laboratory investigations at the presentation showed a normal complete blood count. A comprehensive metabolic panel showed an elevated blood glucose level of 130 mg/dl (70-99 mg/dL), alkaline phosphatase of 225 IU/ml (38-126 U/L), aspartate transferase of 68 U/L (10-42 U/L), and cancer antigen (CA) of 19.9-40.7 U/mL (0-35 U/mL) (Table 1).

**Table 1:** Laboratory investigations at presentation AG: albumin/globulin; CA19-9: carbohydrate antigen 19-9; CEA: carcinoembryonic antigen.

Lab	Value	Range
White blood cells	8.2	4.5-11.0 10*3/uL
Red blood cells	4.73	4.50-5.30 10*6/uL
Hemoglobin	13.5	13.2-17.5 g/dL
Hematocrit	42.7	40.0-53.0 %
Mean corpuscular volume	90.3	80.0-100.0 fL
Mean cell hemoglobin	28.5	25.0-35.0 pg
Mean corpuscular hemoglobin concentration	31.6	31.0-36.0 g/dL
Red cell distribution width	14.2	11.5-14.5 %
Platelet count	236	140-450 10*3/uL
Sodium	142	136-145 mmol/L
Potassium	4.2	3.5-5.2 mmol/L
Chloride	109	96-110 mmol/L
Glucose	130	70-99 mg/dL
Urea nitrogen, blood	14	5-25 mg/dL
Albumin	3.4	3.5-5.0 g/dL
Bilirubin, total	1.7	0.2-1.3 mg/dL
Calcium	9.3	8.5-10.5 mg/dL
Creatinine	1.13	0.61-1.24 mg/dL
Alkaline phosphatase	225	38-126 U/L
Protein, total	6.4	6.0-8.0 g/dL
Aspartate aminotransferase	68	10-42 U/L
AG ratio	1.1	>1.0
Carbon dioxide	26	24-31 mmol/L
Anion gap	7	5-13 mmol/L
Alanine aminotransferase	68	10-60 U/L
Lipase	56	20-55 U/L
CA19-9	40.7	0-35 U/mL
CEA	0.8	0-3 ng/mL
Alpha-fetoprotein, serum	8.0	0-9 ng/mL

**Figure 1:**
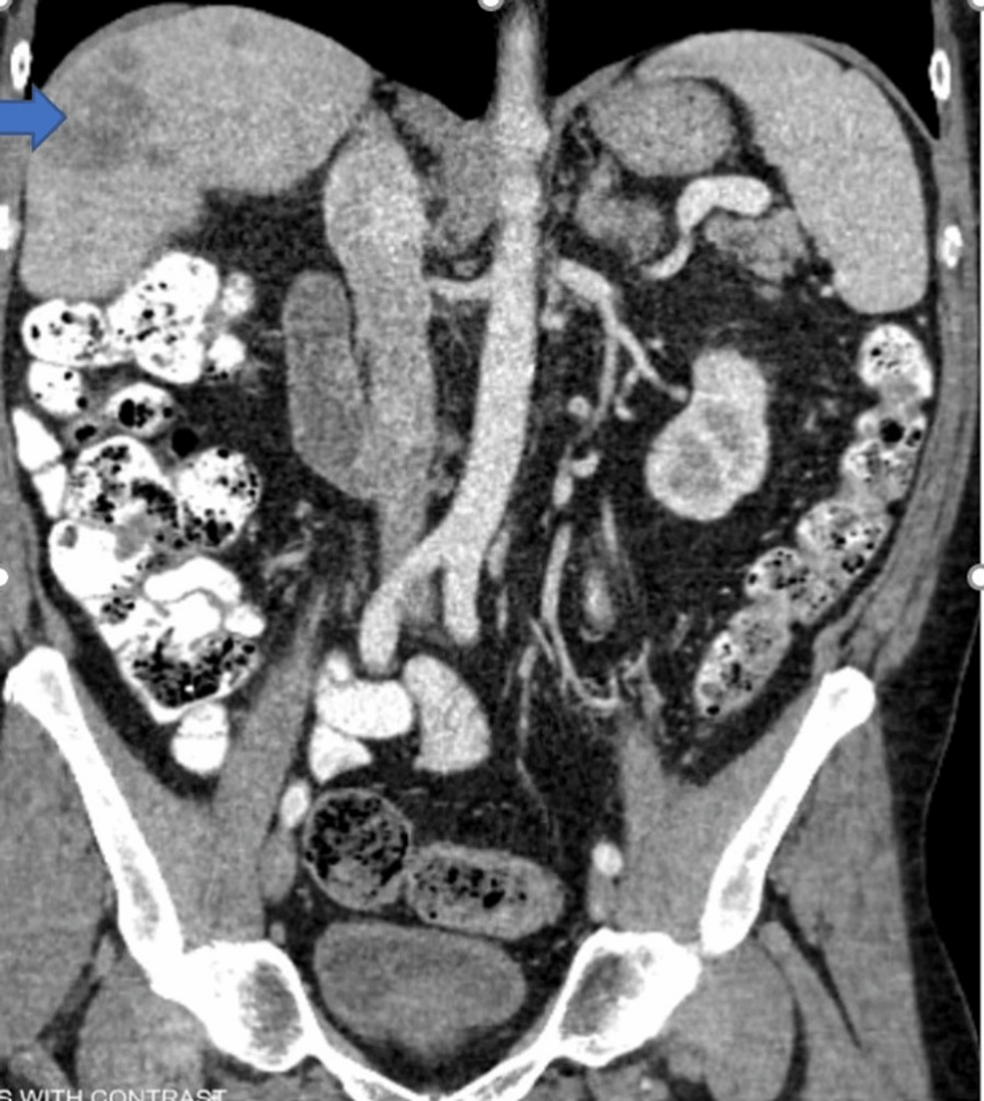
Contrast-enhanced CT of the abdomen and pelvis, coronal view with liver metastasis (blue arrow)

**Figure 2:**
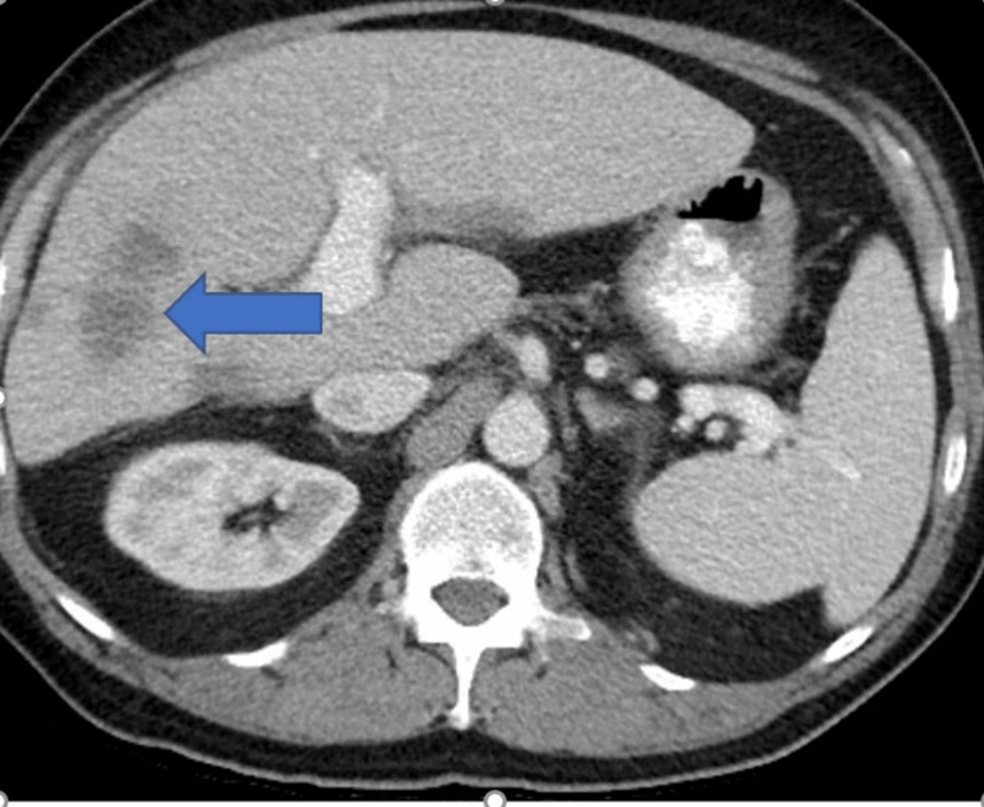
Contrast-enhanced CT of the abdomen (axial view) showing liver metastasis (blue arrow)

**Figure 3:**
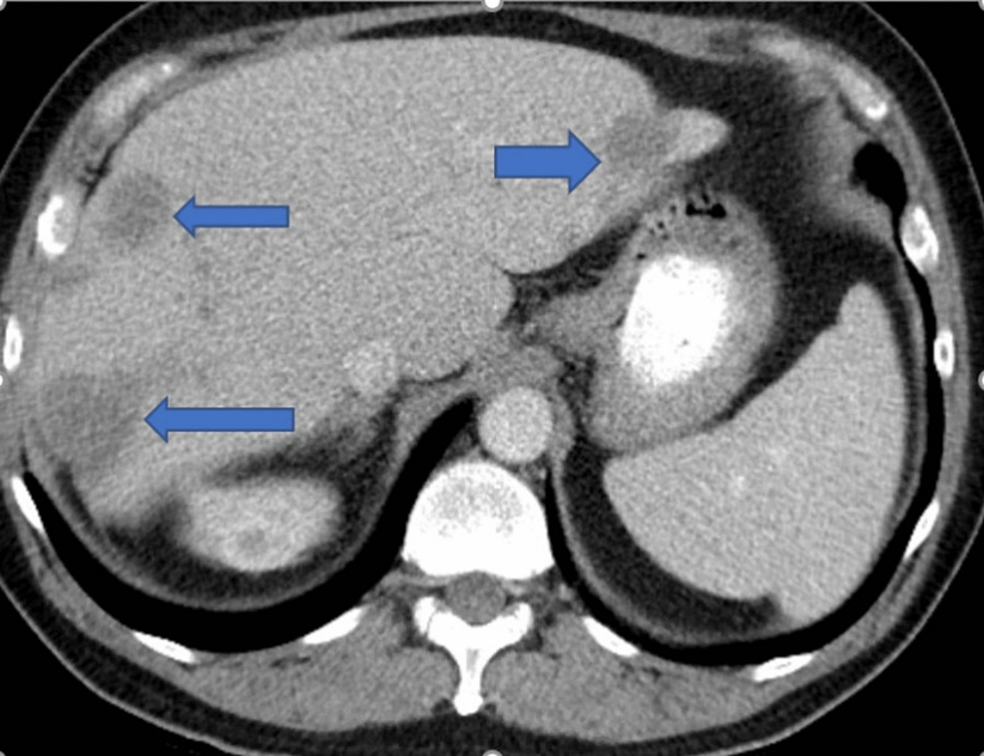
Contrast-enhanced CT of the abdomen (axial view) showing liver metastasis (blue arrow)

Contrast-enhanced computed tomography (CECT) of the chest, abdomen, and pelvis showed multiple liver masses, with the largest measuring 11 x 8.9 cm. Most of the liver masses had central necrosis, suggestive of liver metastases (Figures 1-3).

He had a focal eccentric thickening of the rectum wall and a prostate gland that was slightly bigger than normal, with some parts of the right lateral capsule that were slightly swollen.

**Figure 4:**
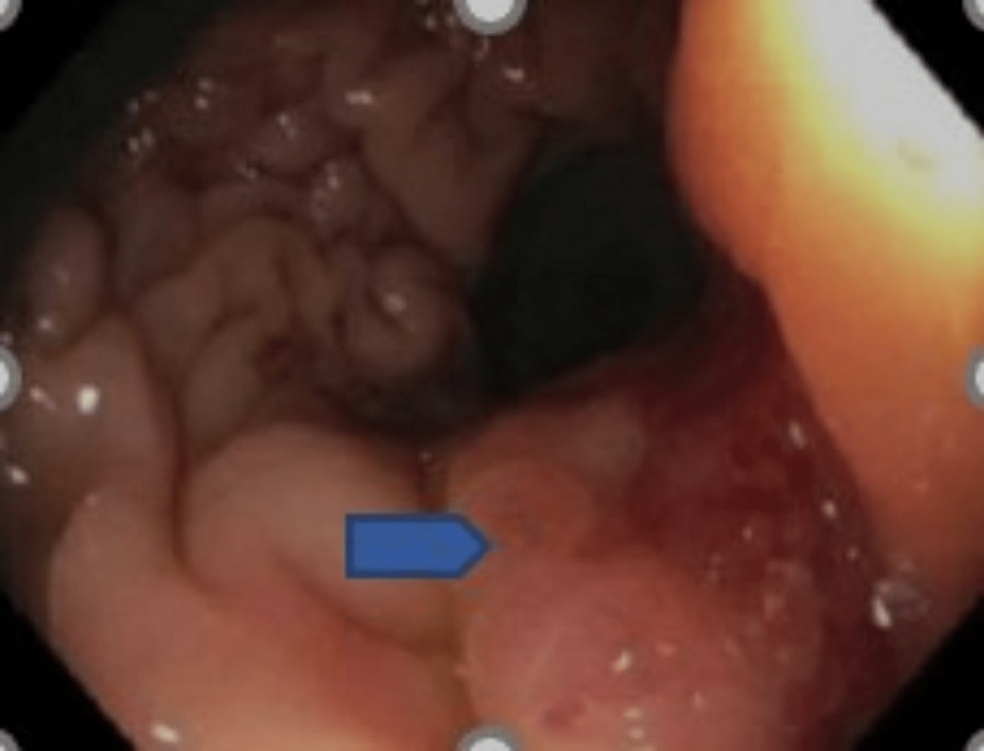
Colonoscopy images from the rectum showing flat mass

**Figure 5:**
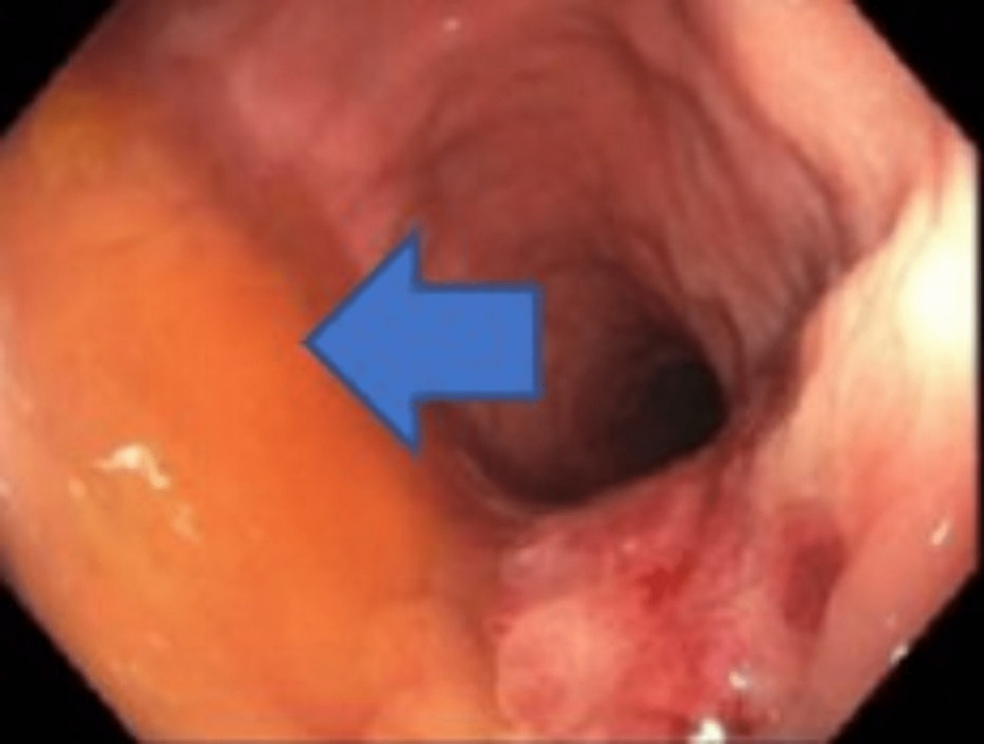
Colonoscopy images of the rectum showing a flat rectal mass

The initial investigations were suggestive of colorectal carcinoma with liver metastasis. A liver biopsy and colonoscopy were done, which revealed a polyp and a flat rectal mass, raising further concerns for rectal carcinoma (Figures 4, 5).

Esophagogastroduodenoscopy showed mild gastritis. Histopathological examination of the liver biopsy specimen showed a metastatic, poorly differentiated carcinoma. Immunostains showed tumor cells positive for NKX3, raising the possibility of prostatic primary. However, prostate-specific antigen (PSA) and prostate-specific acid phosphatase (PSAP) were negative. Poorly differentiated carcinoma was compatible with prostatic origin. Immunohistochemical stains yielded the following results in tumor cells supporting the diagnosis: positive for CAM 5.2 and NKX3.1; negative for CK 7, CK 20, CDX2, chromogranin, synaptophysin, CD56, PAX8, p40, and PSA. The Ki-67 proliferation index was 80%. On further genomic analysis, the tumor was found to be programmed death-ligand 1 (PD-L1) positive, BRAF negative, microsatellite instability (MSI) stable, KRAS negative, and epidermal growth factor receptor (EGFR) negative, and on the cancer type identification, it was 96% compatible with prostate adenocarcinoma stage IV with metastases to liver and rectum. The patient was started on abiraterone hormone therapy, and a positive response has been noted.

## Discussion

Prostatic adenocarcinoma is a slow-growing tumor. It is very unusual to have an initial presentation with liver and rectal metastasis and no other urinary or rectal complaints. Some population-based studies have reported only 10% of men with metastatic prostate carcinoma (MPC) have liver metastasis [2]. Very few cases start with metastasis in the liver, and their outlook is not good [3-5]. The median survival time for patients with liver metastases was 13.5 months (95% CI: 12.7 to 14.4 months) [6]. Also, even though the prostate and rectum are close, prostatic carcinoma rarely spreads to the rectum because of the strong Denonvilliers' fascia that wraps around the prostate. Rectal metastasis by prostate cancer was found in 4% of patients during autopsy [7]. Rectal invasion by prostate cancer may occur via iatrogenic seeding during a transrectal needle biopsy, direct invasion, or lymphatic metastasis into the rectum because they have some common lymphatic drainage [8]. A small retrospective study showed that 4.5% of rectal adenocarcinoma cases were eventually identified as MPC within the positive perirectal lymph nodes [9].

The standard of care for MPC continues to be androgen deprivation therapy (ADT). Most patients with MPC have a high serum PSA level, unlike ours. Usually, undifferentiated and aggressive prostate cancer is associated with low serum levels of PSA and accounts for less than 1% of all patients with metastatic prostate cancer [10]. In these cases, ADT has been relatively ineffective, and the progression is fast. Therefore, the prognosis is generally poor compared to others.

## Conclusions

Prostate cancer is a common malignancy that metastasizes commonly to the lungs, bone, and liver. This being said, obtaining a proper histopathology evaluation is essential before developing a treatment plan for any malignancy. Presentations at the superficial level can be deceiving, as they were initially in this case, presenting with a rectal mass with liver metastasis. Prostate adenocarcinoma with distant metastasis to the liver and colon or rectum is associated with poor outcomes, even though the outcome of other prostate cancers is good. Abiraterone is a new addition to ADT, showing promising results.
